# Predictors of non-obstructive coronary slow flow in poorly controlled type 2 diabetes mellitus: a cross-sectional study

**DOI:** 10.1186/s12872-024-03738-w

**Published:** 2024-02-01

**Authors:** Moataz Ali Hasan Ali Elsanan, Islam Hussein Hassan Hussein Tahoon, Ghada Ibrahim Mohamed, Ibtesam Ibrahim El-Dosouky, Islam Elsayed Shehata

**Affiliations:** Department of Cardiology, Faculty of Medicine, Zagazig University, Sharkia Governorate, Zagazig, 44519 Egypt

**Keywords:** Coronary slow flow, Diabetes mellitus, TIMI flow, Coronary artery disease, Neutrophil-to-lymphocyte ratio (NLR)

## Abstract

**Background:**

Coronary slow flow (CSF) can occur due to various factors, such as inflammation, small vessel disease, endothelial dysfunction, and inadequate glucose control. However, the exact pathological mechanisms behind CSF remain incompletely understood.

The objective of this study was to identify the risk factors associated with slow coronary flow in individuals with Type 2 Diabetes Mellitus (T2DM) who have non-obstructive coronary artery disease (CAD) and experience CSF.

**Methods:**

We conducted a prospective cohort study involving 120 patients with T2DM who were referred for invasive coronary angiography due to typical chest pain or inconclusive results from non-invasive tests for myocardial ischemia. Using a 2 × 2 design, we categorized patients into groups based on their glycemic control (adequate or poor) and the presence of CSF (yes or no), defined by a TIMI frame count > 27. All patients had non-obstructive CAD, characterized by diameter stenosis of less than 40%. We identified many variables associated with CSF.

**Results:**

Our investigation revealed no significant differences in age, sex, family history of coronary artery disease, ECG ischemia abnormalities, or echocardiographic (ECHO) data between the groups. In patients with adequate glycemic control, hypertension increased the risk of CSF by 5.33 times, smoking by 3.2 times, while dyslipidemia decreased the risk by 0.142. Additionally, hematocrit increased the risk by 2.3, and the platelet-to-lymphocyte ratio (PLR) increased the risk by 1.053.

Among patients with poor glycemic control, hematocrit increased the risk by 2.63, and the Neutrophil-to-Lymphocyte Ratio (NLR) by 24.6. Notably, NLR was positively correlated with glycemic control parameters in T2DM patients with CSF.

**Conclusions:**

In T2DM patients with CSF, various factors strongly correlate with glycemic control parameters and can be employed to predict the likelihood of CSF. These factors encompass hypertension, smoking, increased body mass index (BMI), elevated platelet count, hematocrit, NLR, PLR, and C-reactive protein (CRP).

**Trial registration:**

Registry: ZU-IRB (ZU-IRB#9419–3-4-2022), Registered on: 3 April 2022, Email: IRB_123@medicine.zu.edu.eg.

## Introduction

Diabetes, especially Type 2 diabetes mellitus (T2DM), is closely associated with coronary heart disease (CHD). Individuals with diabetes face a two- to four-fold higher risk of cardiovascular disease compared to those without diabetes, with cardiovascular disease accounting for a substantial 70% of diabetes-related mortality. Remarkably, individuals with diabetes but without pre-existing coronary artery disease face a mortality risk similar to that of non-diabetic individuals who have experienced past myocardial infarctions (MI) [1].

Diabetes stands as a prominent driver of atherosclerosis, a condition heavily influenced by inflammation and implicated in various cardiovascular events, including coronary artery disease. Notably, there exists a significant connection between Coronary Slow Flow (CSF) and inflammation, with CSF emerging as a marker of endothelial activation and inflammation [2].

The CSF is defined by a late opacification of epicardial coronary arteries without occlusive disease and angiographically by a delayed progression of contrast medium injected into the coronary arteries [3].

CSF is a common angiographic finding, occurring between 1 and 7% of the time in patients receiving diagnostic angiography due to a clinical suspicion of cardiovascular disease. Clinically, this problem mostly affects smokers and young men who have been hospitalized with acute coronary syndrome. The clinical course is challenging, and almost 20% of patients must be readmitted to the coronary care unit due to recurring chest discomfort that affects more than 80% of patients and is most common when they are rest. The most significant finding is that coronary sluggish flow has been linked to abrupt cardiac mortality and life-threatening arrhythmias, perhaps as a result of greater QTc dispersion in these individuals [4]**.**

Studies have revealed elevated plasma concentrations of high-sensitivity C-reactive protein (CRP) and interleukin-6 in CSF patients. Similarly, higher levels of plasma soluble adhesion molecules such as intercellular adhesion molecule-1, vascular cell adhesion molecule-1, and E-selectin have been associated with CSF. Other inflammatory indicators, including red cell distribution width and serum uric acid levels, have also been linked to the presence of CSF. Consequently, aberrations in inflammatory parameters may signify underlying endothelial dysfunction, a factor closely intertwined with CSF development [4].

The Neutrophil-to-Lymphocyte Ratio (NLR) has garnered significant attention as an inflammation marker, with studies demonstrating its association with an elevated risk of coronary artery disease and a poorer prognosis. Recently, investigations have delved into the relationship between diabetes mellitus (DM) and NLR, shedding light on this emerging research field [5]. The escalation in circulating leukocyte numbers plays a pivotal role in the pathogenesis of various stages of atherosclerosis, from its inception and progression to complications such as atherosclerotic plaque rupture, giving rise to diverse cardiovascular disorders. Studies have substantiated that increased leukocyte counts serve as reliable markers of systemic inflammation, with diagnostic and prognostic implications across angina, myocardial infarction, stroke, peripheral vascular disease, and complications stemming from diabetes in both micro- and macrovascular contexts [6].

Hence, the present study endeavors to assess the predictors of coronary slow flow including the novel inflammatory markers among individuals with Type 2 diabetes mellitus.

## Materials and methods

### Ethical considerations

The study protocol received approval from Zagazig University Institutional Review Board (IRB) ZU-IRB (ZU-IRB#9419–3-4-2022) and was conducted in compliance with the 1975 Declaration of Helsinki, as confirmed by the institution’s human research committee’s prior endorsement. Written informed consent was obtained from all participating individuals.

### Study population

This prospective cohort cross-sectional study was conducted at the Cardiology Department, Faculty of Medicine, Zagazig University, Egypt, from April 2022 to November 2022.

### Sample size estimation

Assuming the confidence level was 95%, our population size was 174 patients with margin of errors about 5%, so the ideal sample size will be 120 cases divided into 4 groups 30 patients in each group according to glycemic control and presence or absence of CSF.

### Participant selection

We enrolled a total of 120 patients diagnosed with Type 2 Diabetes Mellitus (T2DM) who were referred for invasive coronary angiography due to typical chest pain or positive or inconclusive results from noninvasive tests for myocardial ischemia. A 2 × 2 design was employed, assigning 30 patients to each group based on two criteria: 1) adequate glycemic control (yes/no) and 2) the presence of coronary slow flow (yes/no), as defined by a TIMI frame count exceeding 27. All enrolled patients exhibited non-obstructive Coronary Artery Disease (CAD), characterized by diameter stenosis below 40%. The primary objective of this study was to investigate the factors contributing to slow coronary flow in patients with T2DM. We identified nearly 10 variables associated with slow coronary flow.

### Demographics

The mean age of the patients included in the study was 56.62 years, with a standard deviation of 8.6 years. The average Body Mass Index (BMI) was 26.46, with a standard deviation of 2.13. Female representation across all groups ranged from 53.3 to 56.7%, while the male percentage ranged from 43.3 to 46.7%. Clinical history, medication regimens, fasting blood glucose (FBG), and Hemoglobin A1c (HbA1c) levels were evaluated.

### Group stratification

**Fig. 1:**
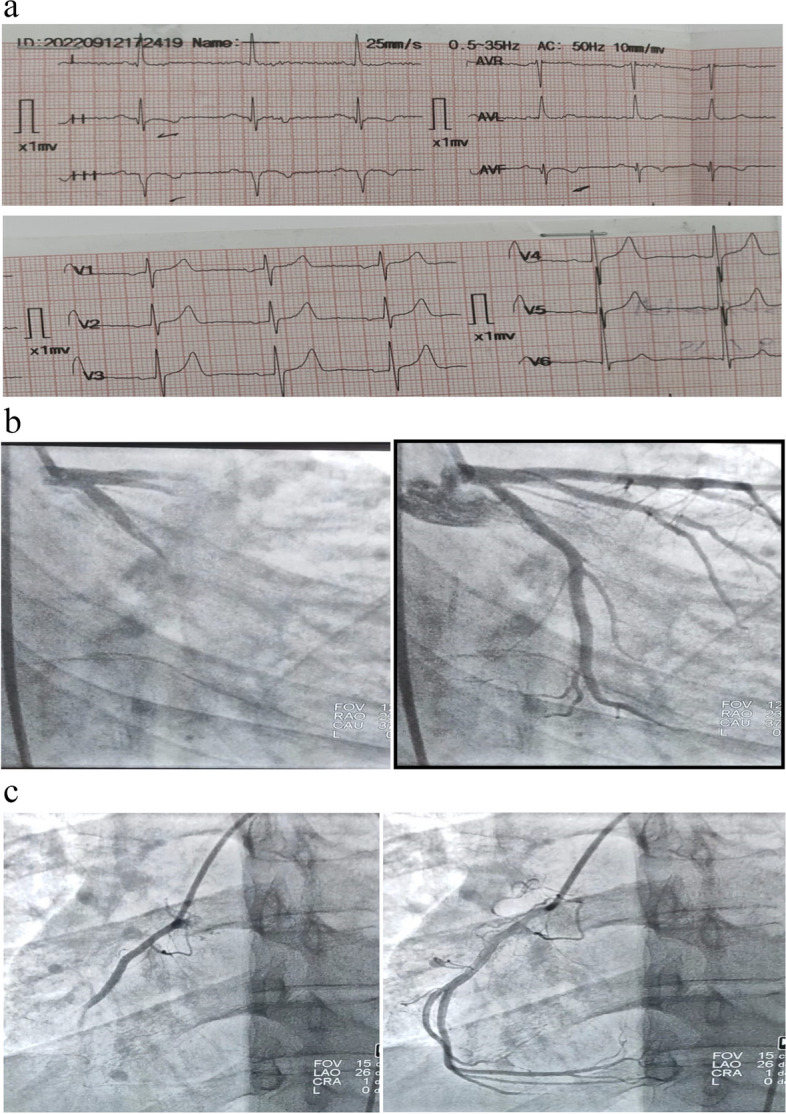
**a** ECG of case from group 4 showing: normal sinus rhythm, heart rate 60 beats per minutes, normal axis, T wave inversion in inferior leads. **b** Coronary angiography of case from group 4: LAO cranial view showing slow flow in LAD and LCX. **c** Coronary angiography of case from group 4: LAO cranial view showing slow flow in RCA

Patients were categorized into four groups based on criteria established by the American Diabetes Association (ADA) for glycemic control [7] and the presence or absence of Coronary Slow Flow (CSF):

- ***Group I:*** Comprised 30 patients with T2DM who demonstrated good glycemic control (HbA1c < 7 & FBG < 126 mg/dl) and had no coronary slow flow.
- ***Group II:*** Included 30 patients with T2DM who exhibited good glycemic control (HbA1c < 7 & FBG < 126 mg/dl) and had CSF.
- ***Group III:*** Consisted of 30 patients with T2DM who had poor glycemic control (HbA1c ≥ 7 & FBG ≥ 126 mg/dl) but lacked CSF.
- ***Group IV:*** Encompassed 30 patients with T2DM who had poor glycemic control (HbA1c ≥ 7 & FBG ≥ 126 mg/dl) and presented with CSF as illustrated in Fig. 1a, b, and c.

### Exclusion criteria

The study excluded individuals with the following conditions: moderate-to-severe valvular heart disease, prosthetic heart valves, bundle branch block, atrial fibrillation, paced rhythm, atrioventricular block, restrictive, hypertrophic, or dilated cardiomyopathies, congenital heart disease, coronary artery ectasia, prior history of myocardial infarction, Type 1 Diabetes Mellitus, uncontrolled hypertension, hyperthyroidism, hypothyroidism, and malignancy.

### Indications for coronary angiography

Coronary angiography was indicated for patients exhibiting typical chest discomfort or yielding positive or inconclusive results from noninvasive assessments for myocardial ischemia. If a noninvasive risk assessment suggested a high-risk event, coronary angiography was performed [8].

### Measurements

- **Patient Information and Risk Factors Assessment:**

- Comprehensive history taking and thorough clinical evaluation included collecting data on age, gender, history of hypertension, medications, smoking status (former, current, never), and other cardiovascular disease risk factors.
- Weight and height measurements were performed to calculate Body Surface Area (BSA) and Body Mass Index (BMI).

b. Electrocardiogram (ECG):

- Standard 12-lead electrocardiograms were obtained for all patients.

c. **Transthoracic Echocardiography (TTE):**

- All patients underwent Transthoracic Echocardiography (TTE) using a commercially available system (Vivid E9, General Electric, Horten, Norway, 2013) upon admission for coronary angiography.
- Standard imaging was acquired using a 3.5–5 MHz transducer at a depth of 16 cm in both parasternal (long- and short-axis images) and apical (two-, three-, and four-chamber images) views.
- The TTE study adhered to the recommendations of the American Society of Echocardiography and the European Association of Echocardiography. When necessary, measurements were indexed to body surface area.
- Parameters such as ejection fraction, left ventricular end-diastolic and end-systolic volumes were calculated using the biplane Simpson’s method, and resting wall motion abnormalities were assessed [9].

d. **Laboratory Investigations:**

- Blood samples were collected from patients in the morning, between 8:00 a.m. and 10:00 a.m., following an overnight fast of at least 8 hours.
- Laboratory tests included a complete blood count (CBC), fasting plasma glucose (FBG), Hemoglobin A1c (HbA1c), and high-sensitivity C-reactive protein (hsCRP) measurements.
- The Neutrophil-to-Lymphocyte Ratio (NLR) was calculated by dividing the absolute neutrophil count by the absolute lymphocyte count.
- The Platelet-to-Lymphocyte Ratio (PLR) was calculated by dividing the absolute platelet count by the absolute lymphocyte count.

e. **Coronary Angiography:**

- The study enrolled patients with confirmed ischemia (as indicated by a positive stress test on nuclear techniques) who lacked obstructive Coronary Artery Disease (CAD) as rigorously defined by quantitative coronary angiography (QCA).
- Diagnostic criteria for Coronary Slow Flow (CSF) were based on the recommendations of Beltrame [10].
- In the course of invasive coronary angiography performed within our catheterization laboratory (Philips Integris 5000, Netherlands), we employed six French diagnostic catheters, specifically Judkins right and left, to access the right and left coronary arteries. Contrast enhancement was achieved through the manual injection of an ionic contrast agent (ioxitalamic acid; Telebrix-35, 350 mg/ml) at volumes ranging from six to 10 ml at each position. Intracoronary injections of 100–200 μg of nitroglycerine were administered to all patients.
- The evaluation of coronary artery flow was conducted by two cardiologists, blinded to patients’ clinical information, using the Thrombolysis in Myocardial Infarction (TIMI) frame count (TFC) method, as established by Gibson et al. [11]. This method entails measuring cine frames by calculating the difference between the first and distal frames, typically viewed at a rate of 30 frames per second. Distal landmarks were pre-defined for each major coronary artery: the left anterior descending artery’s (LAD) distal bifurcation (referred to as the “whale’s tail” or “pitchfork”) for the LAD, the distal bifurcation of the segment with the longest total distance for the left circumflex (LCX), and the first branch of the posterolateral artery for the right coronary artery (RCA).
- To account for the LAD’s usual greater length compared to other coronary arteries, the TFC of the LAD was divided by 1.7 to obtain the corrected TFC (cTFC). The mean cTFC was calculated by averaging the cTFCs obtained from all three vessels. The criteria for diagnosing coronary slow flow were based on guidelines outlined by Beltrame JF [10], requiring at least one main coronary artery, viewed at 30 frames per second, to exhibit a cTFC value exceeding 27 frames, with no significant stenosis or stenosis less than 40% observed during coronary angiography [12].
- The methodology for TIMI frame count measurement was standardized, including catheter size, contrast agent osmolarity, and injection rate.

### Statistical analysis

All statistical analyses were conducted using SPSS version 26 and Medcalc software. The normality of the data distribution was assessed using the Kolmogorov-Smirnov test, confirming that the data were normally distributed. Continuous data were presented as means along with their respective standard deviations, while categorical data were reported as events and percentages.

- For comparing means among more than two groups, the One-Way Analysis of Variance (ANOVA) was employed.
- To compare means between two groups, the Independent t-test was utilized.
- For comparisons involving categorical variables, the Chi-square test or Exact Fisher test was applied.

Associations between variables were assessed as follows:

- Pearson correlation was used to evaluate associations between two continuous variables.
- Phi correlation was employed to measure associations between two categorical variables.
- Point biserial correlation was used to evaluate associations between dichotomous and continuous variables.

The risk factors associated with coronary slow flow were determined through binary logistic regression analysis. This analysis was adjusted for baseline characteristics, including glycemic status, age, gender, and smoking status.

Subsequently, a multivariate analysis was conducted to further investigate the identified risk factors.

The accuracy of predictors for coronary slow flow was evaluated using Receiver Operating Characteristic (ROC) curve analysis and the Kappa test.

In all statistical tests, a significance level of less than 5% (*p* < 0.05) was considered as the threshold for statistical significance.

## Results

The findings of this study are as follows:

- The presence of hypertension increased the incidence of coronary slow flow by 5.33 times in patients with good glycemic control compared to non-hypertensive patients (Table 1).
- Smoking was associated with a 3.2 times higher likelihood of coronary slow flow compared to non-smokers (Table 1).
- Dyslipidemia was found to reduce the likelihood of coronary slow flow by 0.142.
- An increase in hematocrit was associated with a 2.3 times higher incidence of coronary slow flow, and an increase in Platelet-to-Lymphocyte Ratio (PLR) increased the likelihood of coronary slow flow by 1.053 (Table 1).
- In patients with poor glycemic control, an increase in hematocrit was associated with a 2.63 times higher incidence of coronary slow flow, and an increase in Neutrophil-to-Lymphocyte Ratio (NLR) was linked to a 24.6 times higher incidence of coronary slow flow (Table 1).

**Table 1:** Demographic data of all groups

		Group I (*N* = 30)	Group II (*N* = 30)	Group III (*N* = 30)	Group IV (*N* = 30)	*p-*value
**Age (year) mean ± SD**		56.77 ± 7.81	56.57 ± 8.92	56.50 ± 8.96	56.63 ± 9.08	0.99
**BMI (kg/m**^**2**^**) mean ± SD**		23.0 ± 0.7	27.3 ± 1.8	24 ± 1.3	27.3 ± 1.5	*p*_1_*, p*_2_*, p*_*3*_*, p*_4_*, p*_6_ < 0.05 *p*_5_ > 0.05
**Sex**						
**Female**	*N (%)*	16(53.3%)	16(53.3%)	17 (56.7%)	16(53.3%)	0.992
**Male**	*N (%)*	14(46.7%)	14(46.7%)	14(46.7%)	13 (43.3%)	
**Smoking**	*N (%)*	8(26.7%)	18(60.0%)	9 (30.0%)	20 (66.7%)	0.002
**Familyhistory of premature CAD**	*N (%)*	6 (20.0%)	6(20.0%)	7 (23.3%)	6(20.0%)	0.985
**Hypertension**	*N* ***(%)***	7 (23.3%)	22 (73.3%)	9 (30%)	20 (66.7%)	0.006
**Dyslipidemia** **(LDL > 100)**	*N* ***(%)***	20 (66.6%)	7 (23.3%)	21 (70%)	10 (33.3%)	0.001
**Ischemic changes**	*N* ***(%)***	14(46.7%)	16(53.3%)	14(46.7%)	17(56.7%)	0.852
**Hemoglobin**		12.83 ± 0.46	12.86 ± 0.49	12.82 ± 0.5	12.59 ± 0.49	0.29
**Hematocrit**		39.1 ± 0.7	42.2 ± 2.5	39.5 ± 1.0	42.4 ± 2.0	*p*_2_, *p*_3_ > 0.05 *p*_1_, *p*_4_ = 0.05
**Platelets**		219.7 ± 14.6	256.9 ± 19.5	211 ± 22.1	213.3 ± 21.1	*p*_2,_ *p*_4_ > 0.05 *p*_1_, *p*_3_ < 0.05
**Leukocytes**		7.8 ± 1.5	8.5 ± 1.4	8.1 ± 1.3	9.3 ± 1.1	*p*_2_ > 0.05 *p*_1_, *p*_3_,*p*_4_ < 0.05
**Neutrophil**		3.6 ± 0.6	4.6 ± 1.9	4.4 ± 1.3	6.5 ± 1.6	*p*_1_, *p*_2_, *p*_3,_ *p*_4_ < 0.05
**Lymphocyte**		2.0 ± 0.4	1.9 ± 1.0	2.1 ± 0.6	1.7 ± 0.6	0.215
**PLR**		119 ± 33.2	150.2 ± 38.6	107.3 ± 33.8	140.7 ± 58	*p*_1,_ *p*_3_, *p*_4_ < 0.05 *p*_2_ > 0.05
**NLR**		2 ± 0.6	2.6 ± 1.3	2.3 ± 1.1	4.3 ± 2.1	*p*_3_, *p*_4_ < 0.05 *p*_1_, *p*_2_ > 0.05
**CRP**		4.2 ± 0.9	7.8 ± 1.1	4.2 ± 1.6	7.9 ± 1.1	*p*_1,_ *p*_3_, *p*_4_ < 0.05 *p*_2_ > 0.05
**EF**		58.4 ± 5.24	57.53 ± 4.7	57.9 ± 5.09	58.4 ± 5.24	0.9
**HbA1C**		5.8 ± 0.2	6.1 ± 0.4	9.3 ± 0.7	9.6 ± 1	*p*_2_, *p*_3_, < 0.05 *p*_1,_ *p*_4_ > 0.05
**FBS**		104 ± 6.2	110 ± 8.5	252.6 ± 42	271.3 ± 53.1	*p*_1_ = 0.6 *p*_2_, *p*_3_, *p*_4_ = 0.05

*BMI* Body mass index: *CAD* Coronary artery disease

*p*_1_indicate the difference between Group I and Group II

*p*_2_indicate the difference between Group I and Group III

*p*_3_indicate the difference between Group I and Group IV

*p*_4_indicate the difference between Group II and Group III

*p*_5_indicate the difference between Group II and Group IV

*p*_6_indicate the difference between Group III and Group IV

### Predictive values

- The Platelet-to-Lymphocyte Ratio (PLR) had a sensitivity of 81.67% and specificity of 90.9% as a predictor of coronary slow flow in patients with good glycemic control (groups I and III). The cutoff point for PLR was greater than 114.3 (Tables 2 and 3).
- Hematocrit had a sensitivity of 63.33% and specificity of 81.82% as a predictor for coronary slow flow in patients with good glycemic control. The cutoff point for hematocrit was greater than 41.2 (Tables 2 and 3).
- Body Mass Index (BMI) had a sensitivity of 55% and specificity of 90.9% as a predictor for coronary slow flow in patients with good glycemic control. The cutoff point for BMI was greater than 27 (Tables 2 and 3).
- Neutrophil-to-Lymphocyte Ratio (NLR) had a sensitivity of 90% and specificity of 62.5% as a predictor for coronary slow flow in poorly controlled glycemic patients (groups II and IV). The cutoff point for NLR was greater than 2.1 (Tables 2 and 4).
- C-reactive protein (hsCRP) was statistically significant lower in patients without coronary slow flow (Group I, Group III) than patients with coronary slow flow (Group II, Group IV) (*p* = < 0.001) (Table 2).

**Table 2:** Multivariate predictors of CSF for the entire cohort

	Crude odds ratio (OR)			Adjusted odds ratio (aOR^a^)		
	OR	95% CI	*p*-value			
**Age**	1.000	0.953–1.049	0.990	–	–	–
**Sex (female)**	1.144	0.498–2.631	0.751	–	–	–
**Hypertension**	4.667	1.906–11.426	**0.001**	4.638	1.892–11.36	**0.001**
**Smoking**	3.500	1.44–8.5	**0.006**	3.516	1.4–8.559	**0.006**
**BMI**	7.599	2.92–19.714	**< 0.001**	7.31	2.83–18.877	**< 0.001**
**Dyslipidemia**	0.182	0.075–0.443	**< 0.001**	0.177	0.072–0.434	**< 0.001**
**FBS**	2.4	1. 6–6.5	0.01	2.1	1.45–5.7	0.004
**HbA1c**	1.9	1.44–7.5	0.003	1.25	1.1–5.4	0.007
**Family history**	0.821	0.305–2.213	0.697	–	–	–
**CRP**	6.413	2.827–14.545	**< 0.001**	14.220	3.7–54.005	**< 0.001**
**NLR**	1.864	1.149–3.023	**0.012**	1.896	1.166–3.083	**0.010**
**PLR**	1.023	1.01–1.036	**0.001**	1.023	1.010–1.036	**0.001**
**Lymphocyte**	0.820	0.509–1.320	0.413	–	–	–
**Neutrophil**	1.299	0.967–1.744	0.082	–	–	–
**Leukocytes**	1.261	0.916–1.735	0.155	–	–	–
**Platelets**	1.04	1.023–1.058	**< 0.001**	1.065	1.038–1.093	**< 0.001**
**Hematocrit**	2.456	1.673–3.607	**< 0.001**	2.454	1.670–3.607	**< 0.001**
**Hemoglobin**	2.972	1.172–7.540	**0.022**	2.990	1.178–7.590	**0.021**
**WMSI**	0.643	0.150–2.746	0.551	–	–	–
**Ejection fraction**	1.031	0.943–1.128	0.503	–	–	–
**ECG**	1.249	0.546–2.859	0.598	–	–	–

*BMI* body mass index, *CRP* C-reactive protein, *NLR* neutrophil-to-lymphocyte ratio, *PLR* platelet-to-lymphocyte ratio: *WMSI* Wall motion score index: *ECG* electrocardiogram

^a^Adjusted for glycemic status, age, sex

**Table 3:** Accuracy of PLR, hematocrit and BMI as predictors for coronary slow flow in good glycemic controlled patients

	Sensitivity	Specificity	Cutoff point	AUC	95% CI	Youden J index	*p* value
**PLR**	81.67%	90.91%	> 114.3	0.894	0.807 to 0.951	0.73	< 0.0001
**Hematocrit**	63.33%	81.82%	> 41.2	0.680	0.568 to 0.779	0.45	0.0232
**BMI**	55%	90.91%	> 27	0.811	0.709 to 0.889	0.46	< 0.0001

*AUC* Area under the ROC curve, *95% CI* 95% confidence interval, *LR* platelet-lymphocyte ratio, *BMI* body mass index

**Table 4:** Accuracy of NLR as predictors for coronary slow flow in poor glycemic controlled patients (Group III and Group IV)

	Sensitivity	Specificity	Cutoff point	AUC	95% CI	Youden J index	*p* value
**NLR**	90%	62.5%	> 2.1	0.804	0.643 to 0.915	0.53	0.0013

*AUC* Area under the ROC curve, *95% CI* 95% confidence interval, *NLR* neutrophil-lymphocyte ratio

### Other associations

- A one-degree increase in Fasting Blood Sugar (FBS) was associated with a 2.4 times higher likelihood of coronary slow flow (Table 2).
- Hemoglobin A1c (HbA1c) was found to increase the likelihood of coronary slow flow by 1.9 times (Table 2).

These results provide insights into the various factors and biomarkers associated with the incidence of coronary slow flow, especially in the context of glycemic control and other risk factors.

## Discussion

The pathophysiological mechanisms underlying Coronary Slow Flow (CSF) remain largely unknown [13]. This study is significant as it sheds light on the relationship between glycemic parameters and CSF in the context of Neutrophil-to-Lymphocyte Ratio (NLR). The practicality of calculating NLR has prompted numerous recent studies in this direction [13–15].

Our study identified several key findings:

- Good glycemic control was associated with a lower incidence of CSF, potentially explained by reduced inflammatory markers leading to improved endothelial function. This observation aligns with Marfella et al.’s suggestion that optimal peri-procedural glycemic control may enhance post-PCI outcomes by mitigating oxidative stress and inflammation [16].
- Various risk factors and laboratory findings were found to be elevated in patients with CSF compared to those without CSF, making them potential predictors of CSF. These factors include hypertension, smoking, increased BMI, hematocrit, Platelet-to-Lymphocyte Ratio (PLR), and C-reactive protein (CRP).
  Notably, previous studies have yielded mixed results regarding the association between CSF and traditional atherosclerosis risk factors:
- Fineschi et al. [17] and Hawkins et al. [18] found no significant differences in traditional risk factors between CSF and non-CSF patients, possibly due to the high prevalence of risk factors in the general population, which might have obscured distinctions.
- The relationship between hypertension and CSF remains controversial. In our study, hypertension was more prevalent in CSF patients, increasing the incidence of CSF by 4.66 times. This concurs with Sanati et al.’s assertion that hypertension is a strong predictor of CSF. Sanghvi et al. [19] also supported our findings. However, Altun et al. [20] and Mahfouz et al. [21] reported no significant differences, potentially due to variations in sample selection.
- Dyslipidemia, a known cause of atherosclerotic coronary artery disease, was less prevalent in CSF patients in our study, decreasing the incidence of CSF. This corresponds with Mukhopadhyay et al.’s findings. Contrarily, Sanghvi et al. [19] reported higher dyslipidemia in CSF patients, while Altun et al. [20] found no significant differences. This discrepancy may be due to differences in the presentation of epicardial coronaries versus microvascular dysfunction.
- Smoking was strongly associated with CSF in our study, increasing the incidence by 3.5 times. This is consistent with Mukhopadhyay et al. [22] and Selcuk et al. [23]. However, Nurkalem et al. [24] and Gunes et al. [25] reported conflicting results, possibly attributed to regional variations in smoking rates.
- Our study observed a higher BMI in CSF patients, supported by Yilmaz et al. [2], Mukhopadhyay et al. [22], and Gunes et al. [25]. In contrast, Mahfouz et al. [21] found no significant BMI differences, while Sanati et al. [26] reported lower BMI in CSF patients. Obesity may contribute to endothelial dysfunction, potentially explaining our findings.
- Elevated high-sensitivity C-reactive protein (hsCRP) levels were observed in CSF patients in our study, corroborating Madak et al. [27] and Mahfouz et al. [21]. This underscores the role of inflammation in the pathogenesis of CSF. Li et al. [28] also noted higher inflammatory markers in patients with coronary artery ectasia, affirming inflammation’s role in coronary microvascular involvement. However, Erdogan et al. [29] and Mukhopadhyay et al. [22] found no significant differences, underscoring the ongoing debate surrounding inflammatory markers and CSF.
- Hematocrit levels were significantly higher in CSF patients in our study, indicating increased blood viscosity. This aligns with Mahfouz et al.’s findings [21] and suggests a potential role of blood viscosity in CSF.
- Patients with CSF exhibited higher PLR levels in our study, in line with Oylumlu et al. [30], Çetin et al. [31], and Gomaa et al. [32]. This highlights the significance of inflammatory markers in predicting CSF.

Our study further demonstrated that poor glycemic control increases the incidence of CSF. An increase in Fasting Blood Sugar (FBS) or Hemoglobin A1c (HbA1c) was associated with a higher likelihood of CSF. This aligns with the hypothesis that CSF is linked to endothelial dysfunction, a condition exacerbated by chronic hyperglycemia, which accelerates atherosclerosis development, leading to diffuse coronary artery lesions and worsened clinical outcomes [33].

To support this, Sezer et al. [34] observed microvascular impairment in later stages of diabetes mellitus (DM).

In summary, our study contributes to the growing body of knowledge regarding the associations between glycemic parameters, inflammatory markers, and CSF. It highlights the multifaceted nature of CSF and the need for further research to elucidate its intricate pathophysiology.

### Study limitations

While this study has provided valuable insights, there are certain limitations that should be acknowledged:

1. **Limited Sample Size:** The study was conducted with a relatively small sample size of 120 participants. This single-center study’s sample size limitation may affect the generalizability of the findings. Future studies with larger and more diverse participant groups are warranted to validate the results.
2. **Type 2 Diabetes Exclusivity:** This study exclusively focused on individuals with type 2 diabetes mellitus (T2DM). It is essential to recognize that diabetes is a heterogeneous condition, and further research should encompass both type 1 and type 2 diabetes patients to gain a more comprehensive understanding of the relationship between diabetes and coronary slow flow.
3. **Exclusion of Long-term Medications:** The study did not account for the long-term medications used by the participants. Some medications may potentially influence coronary flow, and their exclusion from the investigation may limit the ability to assess their impact accurately. Future research should consider the inclusion of medication history to provide a more comprehensive assessment of the pharmaceutical effects on coronary slow flow.
4. **Short-Term Study:** This study primarily focused on short-term observations, potentially overlooking the effects of long-term drug usage on coronary flow. To gain a deeper understanding of how pharmaceuticals can impact coronary slow flow, conducting long-term studies that incorporate medication usage over extended periods is recommended.

### Clinical implication

The findings of this study have several clinical implications:

- Patients with comorbidities such as hypertension, a history of smoking, increased BMI, elevated hematocrit values, high Neutrophil-to-Lymphocyte Ratio (NLR), high Platelet-to-Lymphocyte Ratio (PLR), and increased C-reactive protein (CRP) levels are at a heightened risk for the development of Coronary Slow Flow (CSF). These factors can serve as valuable indicators for healthcare professionals to identify patients who may be more susceptible to CSF.

## Conclusions

In summary, this study has shed light on the significant associations between various clinical and laboratory factors and CSF in patients with type 2 diabetes mellitus (T2DM). The key conclusions drawn from this research are as follows:

- In T2DM patients with CSF, multiple factors are strongly correlated with glycemic control parameters. These factors encompass hypertension, smoking, increased Body Mass Index (BMI), elevated hematocrit levels, high Neutrophil-to-Lymphocyte Ratio (NLR), high Platelet-to-Lymphocyte Ratio (PLR), and elevated C-reactive protein (CRP) levels. These factors collectively contribute to the prediction of CSF in this patient population.

### Recommendations

Building on these findings, several recommendations for future research and clinical practice emerge:

- There is a need for larger-scale, multicenter studies to corroborate and strengthen the validity of the results obtained in this study. Expanding the scope of research to encompass a broader and more diverse patient population can enhance the generalizability of the findings.
- Long-term studies that encompass the medication history of patients are highly recommended. Investigating the relationship between pharmaceutical agents and the occurrence of CSF over extended periods can provide deeper insights into the influence of drugs on coronary flow.

## Data Availability

Our data used to support the findings of this study are available from the corresponding author upon request.

## Abbreviations

ADA: American diabetes association
CAD: Coronary artery disease
CGC: Conventional glycemic control
CRP: C-reactive protein
CSF: Coronary slow flow
cTFC: Corrected TIMI frame count
DM: Diabetes mellitus
DPP4: Dipeptidyl-peptidase 4
ECG: Electrocardiogram
ECHO: Electrocardiography
FBS: Fasting blood sugar
FC: Thrombolysis in myocardial infarction frame count
NLR: Neutrophil-to-lymphocyte ratio
PLR: Platlet-to-lymphocyte ratio
